# Mentalizing in the context of Mentzos’ dilemma–on the use of implicit work in the treatment of non-affective psychosis

**DOI:** 10.3389/fpsyt.2023.1229113

**Published:** 2023-07-17

**Authors:** Anna-Lena Bröcker, Dorothea von Haebler, Günter Lempa, Christiane Montag

**Affiliations:** ^1^Department of Psychiatry and Neurosciences, Campus Charité Mitte, Charité–Universitätsmedizin Berlin, Corporate Member of Freie Universität Berlin and Humboldt Universität zu Berlin, Berlin, Germany; ^2^International Psychoanalytic University Berlin, Berlin, Germany; ^3^Psychotherapy Practice, Munich, Germany

**Keywords:** mentalizing, metacognition, psychosis, schizophrenia, dilemma, implicit, explicit, psychotherapy

## Abstract

Current approaches to the treatment of non-affective psychosis include elements of mentalization-based treatment and the potential in enhancing mentalizing capacity in this patient group has been widely emphasized. This article presents the “psychotic identity dilemma”, a concept by Stavros Mentzos, and a therapeutic approach considering this concept as a valuable complementary addition to these treatments. The idea of a dilemma between closeness and distance, which in itself cannot be represented mentally at first, helps to respond to specific needs of patients with psychotic disorders by placing the treatment focus on fundamental interpersonal processes. Following this train of thought, this article attempts to shed light on the importance of *the “real relationship” between therapist and patient as well as the exploration of the “here and now”,* especially at the beginning of psychotherapeutic treatment. Two treatment modes are suggested, one characterized by the *experience of interpersonality* within the therapeutic relationship and a second one characterized by the *reflection of interpersonal phenomena*. These modes are framed by Stavros Mentzos’ concept of an identity dilemma. We describe how mentalizing first needs to be addressed *implicitly* in a tolerable, exemplary relationship in which closeness and distance are regulated based on the therapist’s countertransference, then *explicitly*. A series of interventions are described, which allow for moments of shared attention, promote intentionality and contingency and, later in the course of therapy, help to integrate experiences into narratives.

## Introduction

Because of their clinical relevance and empirical evidence (1), mentalization-based treatments have received a lot of attention in recent decades. While mentalization-based therapy (MBT) was initially designed for the psychodynamic treatment of personality disorders (2), nowadays mentalization-based approaches have found their way into the treatment of other disorders including psychosis (3, 4). Mentalizing is not easily differentiated from neighboring concepts. In fact, it empirically overlaps with concepts such as synthetic metacognition (5–7), which describes the ability to reflect on oneself and others (in terms of cognition, emotions and intentions) and to integrate this mental knowledge into increasingly complex narratives (of self and others) (8, 9). In contrast to mentalizing, the concept of synthetic metacognition does not offer an explanation as to how the respective abilities form in childhood. It is therefore considered as a rather descriptive approach, which can be complemented by mentalization theory (10). Metacognitive deficits have been found to explain many characteristic features of patients with schizophrenia, such as misattributions of others’ mental states (11), lack of self-reflection (12), or difficulties in establishing a trusting therapeutic relationship (13), to name a few. They have also been found to be positively associated with functioning and psychopathology in this patient group (14).

As a result, various psychotherapeutic approaches aim to promote mentalizing or metacognition; both direct adaptations of MBT (3, 4, 15, 16) and further approaches (17–20). We aim to critically reflect on how specific they are with regard to psychosis. What distinguishes people with schizophrenia from other patients experiencing psychotic symptoms, e.g., in the context of post-traumatic stress or severe impairments of personality function? How can the therapeutic approach be refined and even better adapted to the needs of patients with “primary” psychoses? Especially in the initial phase of treatment; how can the difficulty to engage patients and their often critical ambivalence toward treatment be conceptualized and managed?

In the search for the specific, subjective experience of people suffering from “primary” psychotic disorders, the phenomenological view has contributed significantly to the understanding of psychosis as a disorder of the self (21, 22). An elementary (implicit) perception of the self is a prerequisite for all experience, called “ipseity” (23) or “minimal self” (24). In psychosis, this self-evidence of perceiving, being, and interacting in an environment as well as temporal orientation can get lost (22, 25, 26). The weakening of the pre-reflective sense of self can lead to profound experiences of alienation in relation to one’s own physical or mental processes, but also in relation to interactions with the environment. This includes, in particular, a loss of interpersonal resonance, i.e., the ability to intuitively attune to an interaction partner. Bodily and emotional attunement processes based on pre-reflexive knowledge, also described as intercorporeality (27), are altered during psychosis; schizophrenia has therefore also been described as a disembodiment disorder (28, 29).

According to one strand of psychodynamic thinking, the difficulties of people with psychosis in regulating interpersonal relationships are due to an inability to integrate the unconscious motivational themes of autonomy and dependence, which are considered foreclosed, but not repressed. Foreclosure in this sense is a form of defense that makes any representation impossible, in contrast to repression, where preconscious representations exist and contradictory motives are in principle accessible (30). This incompatibility of autonomy and dependence as the core of vulnerability for psychosis has been associated with a weakening of “ego-boundaries” in both classical writings and contemporary research (31–35). It has also been associated with changes in the constitution of the self [for a summary see Lempa et al. (17)]. We would therefore like to introduce the “psychotic identity dilemma”, a concept by Mentzos (36), as the key concept of this article. When considering this concept, it is necessary to focus primarily on implicit techniques at the beginning of treatment. We postulate that this focus, which is implemented rather intuitively by many therapists, is an “active agent” in the successful treatment of psychosis. The specificity of implicit (versus explicit) mentalizing has already been emphasized in recent work (37). In the case of psychosis, we argue that patients can only improve on first implicit and then explicit mentalizing when the patient’s dilemmatic formation of relationship is addressed in therapy.

## Psychotic identity dilemma

The psychotic identity dilemma (36, 38) builds on the ideas of earlier psychoanalysts that a field of tension exists between need and fear (39), autonomy and dependence (33) or between symbiotic and separate states of self (34). This dilemma is defined by an existential intrapsychic polarity between self-related and other-related tendencies, between closeness and distance, autism and fusion. Mentzos postulates that both biological and biographical factors may contribute to an individual’s difficulty to reconcile or integrate these tendencies (40). This results in a permanent unconscious tension, which is assumed to form a predisposition for psychosis. An affected individual is – unconsciously – tossed back and forth between trying to enter into relationships (with the risk of dissolving ego-boundaries) and trying to gain an identity as a person (with the risk of losing contact with a necessary “Thou”) – both options pose an existential threat. Since the dilemma is thought to form in a developmental phase dominated by pre-symbolic processes, it cannot be represented mentally, and thus cannot become the subject of explicit reflection or interpretation. The challenge of a “dilemmatic” psychic structure in interpersonal situations that require an integration of these polarities can cause existential fears in the patient, which in turn can elicit strong emotions in any person interacting with the patient.

Mentzos’ concept has been incorporated as a central theory in a recent modification of psychodynamic psychotherapy for people with schizophrenia (MPP-S; 17). Here, a distinction was made between the role of the dilemma as a predisposition for psychosis and its actualization in acute phases of the disorder. The dilemma may remain a latent vulnerability as long as compensation of tension is possible, e.g., through autistic withdrawal or through self-sacrifice by over-adaptation in a “symbiotic relationship.” However, if the structural possibilities of regulation are overstrained, as for example in “threshold situations” (e.g., moving out of the parental home), a further breakdown of the integrative capacities of the ego occurs. A solution to the dilemmatic situation comes at a cost of a psychotic loss of shared reality (38).

Mentzos emphasizes the compensatory character of psychotic symptoms and speaks of defense mechanisms that relate to the underlying dilemma. In this understanding, symptom formation is conceived as a functional attempt to maintain an – albeit distorted – connection to the social world and to protect the boundaries between self and others. It cannot be equated with defense mechanisms of a mature mental apparatus, since the dilemma itself cannot be represented and both poles of the dilemma are existentially threatening. However, such a conceptualization helps to understand and acknowledge an interpersonal “function” of symptoms. In persecutory delusion, for example, the proximity to the persecutor can be secured, but the persecutor never becomes too threatening because close contact is avoided due to fear and suspiciousness (41). From Mentzos’ viewpoint (40), specific therapeutic interventions can be derived that address the underlying problem in order to reduce symptoms (by promoting “constructive” closeness versus distance) and that – at least initially – do not rely on verbal-explicit reflection and interpretation.

Taking Mentzos’ dilemma into account may help to prevent the actualization of an acute dilemma, and thus acute exacerbation of psychosis, patient’s withdrawal or other negative sequelae. Allowing for an exemplary, model experience of a tolerable, non-overwhelming, but real and committed “I-Thou” relationship (42) is the main goal of the initial stage of psychodynamic psychotherapy. Symptoms are regarded as an expression of the identity dilemma and are treated on the premise that they will no longer be necessary once the dilemma is defused.

## “Dilemma-sensitive” regulation of the relationship

It is to Mentzos’ credit that he has emphasized the importance of a “real” relationship and thus the importance of implicit coordination processes between patient and therapist (38). Conceptually, the dilemma is assumed to emerge in early developmental phases in which the so-called implicit knowledge about relationships is shaped (43, 44). Fine-tuned interactions between mother and child are embodied and form the basis for later interpersonal interactions. This knowledge is pre-reflexive, i.e., without mental representation, but operates unconsciously into adulthood when people interact with others. The quality of shared experience is crucial for development, though it cannot be abstracted into words. The treatment of psychosis is based on this idea. Since there is no symbolization for an interpersonal dilemma and no possibility to reflect on it, fundamental processes need to mature and the dilemma needs to be defused in a reasonable period of time before representation becomes possible (44, p. 224). To defuse the dilemma, the therapist’s focus is on the therapeutic relationship and shared experiences in *the here and now.* The aim is to create moments of constructive closeness and constructive distance that reduce interpersonal anxiety, by using implicit techniques.

By “moving along” (44, 45), therapist and patient aim to (re)gain the ability of experiencing, perceiving reality with the ego intact. The therapist’s focus is always on the intersubjective field; an intrinsic need for contact is the basic premise of a therapeutic situation and makes the shared experience so meaningful. However, the explicit focus of the session can often lie on a physical “third,” and intersubjective topics might be largely avoided. Talking about basic topics might be necessary to reduce interpersonal fear and set the stage for a relationship. By welcoming any issue the patient brings in and by cautiously encouraging a joint, careful exploration of thoughts and feelings as well as details, context or implications, the shared reflective space can be gradually largened. Through many small implicit regulative circles the intersubjective field is constantly shaped and at best enlarged. “Moving along” is by definition an implicit process, but one that opens up directions that can later be explored explicitly. However, it is crucial that the therapist avoids actualizing the dilemma, by on the one hand, asking too demanding questions (being too intrusive) or, on the other hand, by not showing any curiosity (being too absent).

Stern (44, 46) described so-called “now-moments” that occur unexpectedly and mark an interruption in the moving along within a therapeutic process [“nonlinear jumps” (46, p. 304)]. An interpersonal encounter happens with a strong affective quality–dealing with it “authentically” and constructively, i.e., in our understanding mitigating the high tension of the contact and still maintaining a connection, can lead to so-called “moments of meeting” that change the relationship in a lasting way. This change represents a new state of intersubjectivity. Two separate individuals meet, pause, and continue down a (changed) path. Repeated “moments of meetings” expand the interpersonal field and alter implicit relational knowing. In terms of Mentzos’ dilemma, these encounters can lead to the experience of a new kind of relationship, a “rewriting” of the past: this means that a separate identity and a relationship do not have to be mutually exclusive. Such moments do not need to be interpreted or verbalized to be effective; rather, they run the risk of being truncated by this and not pertaining to real experience. They should be experienced in “real time” (44, p. 226).

## Implicit techniques to promote mentalizing

Although the promotion of mentalizing in psychotherapy has its roots in developmental psychology, it has traditionally been understood as aimed at developing explicit, conscious reflection on the mental states of the self and others. Recently, representationalist accounts of social cognition that focus on theory- or simulation-based third-person perspectives have been complemented by enactivist, interaction-based or embodied mentalization approaches. Since the psychotic dilemma is a non-representable state, thinking about mentalizing needs to be extended to its embodied forms (47–49).

Observing the melody of speech and the rhythm of turn-taking, as well as carefully encouraging kinaesthetic interactions and thus emotions, can help build “primordial empathy” (50). Eye contact, mimic expression, and body posture can be synchronized in a very cautious (and mostly intuitive) way, bearing in mind that resonance can indicate interpersonal closeness, but can also become threatening. Any intrusiveness as well as empathic “overexcitement” should be avoided, especially in the case of aversive emotions, as they can limit the ability to mentalize and increases (interpersonal) distress. The therapist can use interruptions in synchrony and bring about subtle changes in voice to mark distance and regulate potential dilemmatic escalations. In addition to promoting shared experiences and synchrony, working on “ego-boundaries” or demarcations is equally important. The therapist may casually mark “like me” or “other than me” situations. Intentional acts of the patient as such should be appreciated and not be discouraged.

The main goal in the initial phase of treatment is to create a tolerable “real relationship” (45) between therapist and patient that will serve as an example for later relationships. This basically means reacting to the underlying dynamics of an (assumed) dilemma. Therefore, the therapist should regulate the “appropriate dose” of interpersonal contact and the “emotional temperature” during sessions. This can be done by asking for feedback directly, but also by adjusting the speaking time and allowing for changes in session frequency and duration. It is important for the therapist to become visible as a dialogical “Thou” with their own perspective and mental processes, not hiding behind expert knowledge or a particular technique.

Bion (51) introduced the term “negative capabilities” to describe the therapist’s ability to endure doubt, paradox, confusion, or misunderstanding, and to resist the urge to end this state of not knowing too quickly by placing it in interpretive terms or diagnostic categories. This leads to an open, authentic attitude, as it is also known from MBT. With regard to Mentzos’ dilemma, however, each therapeutic action is examined for its potential to mitigate or reactivate the dilemma – it may then be a matter of taking a step back accordingly (17). This may apply to various interpersonal constellations: some patients avoid contact and tend to withdraw or strongly control the conversation and negate the therapist’s existence, while others rather adapt and virtually disappear in the presence of another person.

In acute psychosis, patient and therapist may turn to something “third” (e.g., an everyday occurrence, a hobby, or an external stimulus) to alleviate the interpersonal tension. For this purpose, it can for example be helpful to actually go for a walk together and talk about what you see. Thus, joint attention, the turning to something third, takes place dialogically and physically. It can also be helpful to respond in a “dialogue of action” (52). This involves responding adequately through actions to the other person, who has limited access to verbal representations during psychosis. The therapist attempts to interpret the patient’s actions and forms hypotheses about their origins that are not yet communicable. By acting in a reflective, “responding” manner, escalations are avoided and communication remains possible.

In the developing relationship, mentalization is encouraged as one “moves along” (44, 45). Emotions are addressed and reflected when it seems possible on an interpersonal level. Therapists can also help by vicariously providing their own emotional perspective. Some emotions appear to have been discarded in the process of psychotic symptom formation; these are kept in mind by the therapist as a vanishing point while work is done on the structural capacities to experience and regulate emotions. Implicit work is thus constantly interwoven with explicit interventions. It should be noted that implicit interactions can only partly be regarded as conscious actions of the therapist. Reflection on countertransference or action dialogs can in many cases only take place retrospectively, but represents the therapist’s main instrument for creating favorable conditions for developments of the (body) ego organization.

Ideally, there is a second phase of therapy that focuses on clarifying, interpretive, and confrontational elements of therapy through so-called explicit techniques. An increasingly reflexive approach serves the goal of gradually integrating the experience into one’s own life narrative. This includes reflecting on the causes and conditions of psychosis, exploring its subjective meanings, focusing on the feelings that arise in the therapeutic relationship, and noticing and grieving negative experiences. Building narratives and integrating essential experiences into one’s biography and self-concept is an essential aspect of every psychotherapy. However, this process requires abilities such as decentration and introspection, which are not always accessible for people with psychosis. They may be impaired in situations of high arousal or during acute psychosis or may be limited to certain areas of functioning.

The ability to mentalize should be continuously assessed by the therapist, as should the patient’s tolerance of interpersonal relationships. We argue that an underlying identity dilemma threatens these premises and must first be addressed and mitigated. Only then it becomes possible to intervene explicitly and reflexively. However, focusing a dynamic regulation of closeness versus distance is always relevant and comes to the fore when the dilemma is very present and causes strong anxiety in the patient. Reactivations of dilemmatic experiences are also possible at later stages of treatment or are confined to particular spheres of life. A sequence of two strictly separated phases is therefore ideal-typical. In reality, therapists should always be sensitive and resort to work with the implicit when necessary. A high degree of flexibility is required to alternate between both modes at the patient’s pace, guided by their countertransference.

## Countertransference

Countertransference or co-transference (53, 54) can have an existential quality in the case of psychosis. For example, the patient’s psychotic anxiety may evoke a strong response in the therapist, which, if not adequately reflected upon, may produce unbalanced or even harmful reactions. Reflecting on strong and diffuse feelings in response to a person facing the existential threat of losing their identity, can help to understand the patients’ tendencies to avoid or to control the interaction or to defend themselves. When dilemmatic fears are not explicitly perceived, acknowledged and reflected upon, therapists might unconsciously react inappropriately. They might take all responsibility, give inappropriate personal information, or – on the other hand – become “too technical” and leave the patient to their own devices [for an overview see (17, p. 92ff)]. Another manifestation of the dilemma (in countertransference) can be extreme subtlety or cautiousness or even a desire for fusion and symbiosis in the therapist (55). Sometimes therapists may experience unusual somatic reactions during sessions. Lombardi (56) introduced the term “somatic countertransference” as an indication of mind–body dissociation in the patient, the presence of “asymbolic and pre-symbolic areas of the mind that are deeply embedded in the body” (57, p. 1426). These must first be contained within the therapist’s body before any kind of mentalizing can take place along with the establishment of a “body–mind-contact network” (58).

By constantly “scanning” one’s own emotional reactions and impulses, the therapist may discern indications of repetition of pre-symbolic patterns of interaction, reflect on them, and respond accordingly. Thus, permanent re-actualization of the dilemma and retaliatory attacks by the patient can be avoided (59) and the need for maternal-like care or temporary aversive feelings can become tolerable. In the best case, a calm and helpful climate can be maintained during the session. The ability to perceive and classify countertransference reactions has a relieving and triangulating effect on the interpersonal space. Classification also helps the therapist not to avoid these existential affects, but to reflexively gain space and capacity to respond empathically and without too much anxiety to the patient’s relational offer (17). Consequently, observing, reflecting on and dealing with countertransference is an essential technique in psychodynamic therapy for psychosis.

## Main additions to current modifications of MBT

Mentalization-based therapeutic strategies draw on the one hand from cognitive neuropsychology, which examines the central role of meta-representation, including the theory of mind (ToM), in the manifestation of psychosis (60) and on the other hand from findings in developmental psychology, which emphasize early infant-caregiver interactions and the attachment relationship (61). Current approaches of MBT for psychosis go further and acknowledge the role of embodied mentalizing as a link between sensory-affective signals and cognitive mentalizing (4, 15, 62). Here, the idea is implied that emotional experiences are disconnected from representational states due to anxieties about painful emotions threatening the state of the self. However, the underlying dynamics of interpersonal anxieties between closeness and distance, as described in the dilemma concept, have not been conceptualized before and could be a valuable addition.

Mentzos’ dilemma concept extends the explanatory models for the development of psychosis as a functional, though imperfect attempt to regulate relationship and emphasizes the role of the pre-reflective, motivational themes of identity and dependence. With this in mind, some additions to MBT should be considered. It is crucial to mention that these additions do not touch core principles of MBT, such as a “not knowing” therapeutic stance, treating the patient as an intentional agent, the joint search for subjective meaning, a focus on currently felt affects and a careful adjustment to the patient’s current level of mentalizing (2). Rather, in our understanding, a “dilemma-sensitive” establishment and regulation of the therapeutic relationship – far beyond the cognitive interventions based on it – must always accompany these processes. It is a specific task and mainstay of treatment for patients diagnosed with primary non-affective psychosis, which requires primary attention and sufficient time. With regard to the later phases of therapy, in which reflection and narrative formation gradually come to the fore, the corrective interpersonal experience helps to strengthen the structural basis for the experience of inner and outer reality. In the process of establishing relationship, the therapist can become a supportive and authentic “Thou” who helps to re-constitute reality. This often includes non-social, but concrete aspects of reality, before mentalizing work can become more central.

Fostering epistemic trust as a principle of MBT (63) is important, but still secondary to the therapist’s ability to create a moment-to-moment experiential, “just tolerable” human encounter. Tolerability in the sense of a non-dilemmatic exemplary relationship would not begin with attention to the patient’s mistrust or attachment representation (and the therapist’s respective attitude and interventions), but earlier in the pre-reflective, embodied forms of meeting, comparable to parental embodied mentalizing (37, 64). Regulation at this stage can only take place on the basis of therapist’s countertransference, which allows to perceive the optimal interpersonal “dose”. Spatial distance, bodily and verbal presence, session duration and frequency are adjusted on this basis. Structured therapy elements such as a therapeutic contract or psychoeducation, which have been highlighted as prerequisites for MBT in psychosis (4, 65), could activate the dilemma in one case by the powerful presence of another intentional agent (the therapist), or help triangulate an overwhelming dyadic situation in other cases. We believe that all of these components of therapy can be applied, but should be reflected upon for their impact on the patient’s assumed dilemmatic disposition. Trust in the truthfulness, generalizability and relevance of the therapist’s statements can grow, become conscious, and can increasingly help to reduce epistemic hypervigilance (63). As a result, cognition-based approaches like re-establishing theory of mind and perspective-taking will become more significant.

In summary, we would like to propose to further elaborate the implicit characteristics and techniques of MBT and other therapies for the treatment of patients with non-affective psychosis. We have postulated that in these patients a dilemma of conflicting motives (attachment versus autonomy), which initially cannot be represented mentally, is a characteristic, basal pathomechanism, the consideration of which can provide a valuable background for any other intervention. Implicit techniques should be considered specific here. Our contribution is intended to encourage the exploration of this hypothesis, including potentially elusive processes such as dealing with countertransference or embodied interaction. Our perspective is also intended to contribute to creating an awareness for those patient groups for whom relational functioning is one of the fundamental aspects of their illness and who therefore need sufficient time and space to work on these difficulties in “real time” (44, p. 226).

## Data availability statement

The original contributions presented in the study are included in the article/Supplementary material, further inquiries can be directed to the corresponding author.

## Author contributions

All authors listed have made a substantial, direct, and intellectual contribution to the work and approved it for publication.

## Funding

We acknowledge financial support from the Open Access Publication Fund of Charité – Universitätsmedizin Berlin and the German Research Foundation (DFG).

## Conflict of interest

The authors DvH, GL, and CM published a book on the presented therapeutic approach (MPP-s).

The remaining author declares that the research was conducted in the absence of any commercial or financial relationships that could be construed as a potential conflict of interest.

## Publisher’s note

All claims expressed in this article are solely those of the authors and do not necessarily represent those of their affiliated organizations, or those of the publisher, the editors and the reviewers. Any product that may be evaluated in this article, or claim that may be made by its manufacturer, is not guaranteed or endorsed by the publisher.

## References

[1] Storebo OJ Stoffers-Winterling JM Völlm BA Kongerslev MT Mattivi JT Jorgensen MS. Psychological therapies for people with borderline personality disorder. Cochrane Database Syst Rev. (2020) 2020:CD012955. doi: 10.1002/14651858.CD012955.pub2, PMID: 32368793 PMC7199382

[2] Bateman AW Fonagy P. Psychotherapy for borderline personality disorder: Mentalization- based treatment. Oxford: Oxford University Press (2004).

[3] Brent B. Mentalization-based psychodynamic psychotherapy for psychosis. J Clin Psychol. (2009) 65:803–14. doi: 10.1002/jclp.20615, PMID: 19572277

[4] Weijers J Ten Kate C Viechtbauer W Rampaart LJA Eurelings EHM Selten JP. Mentalization-based treatment for psychotic disorder: a rater-blinded, multi-center, randomized controlled trial. Psychol Med. (2021) 51:2846–55. doi: 10.1017/S0033291720001506, PMID: 32466811 PMC8640364

[5] Hasson-Ohayon I Lysaker PH. The recovery of the self in psychosis: contributions from metacognitive and mentalization based oriented psychotherapy. London: Routledge (2021).

[6] Bröcker AL Zimmermann J Stuke F Just S Bayer S Mielau J. Exploring the latent structure and convergent and incremental validity of the metacognition assessment scale - abbreviated in a sample of patients with non-affective psychosis. J Pers Assess. (2023) 105:100–10. doi: 10.1080/00223891.2022.2048843 35363095

[7] Lysaker PH Carcione A Dimaggio G Johannesen JK Nicolò G Procacci M. Metacognition amidst narratives of self and illness in schizophrenia: associations with neurocognition, symptoms, insight and quality of life. Acta Psychiatr Scand. (2005) 112:64–71. doi: 10.1111/j.1600-0447.2005.00514.x, PMID: 15952947

[8] Hasson-Ohayon I Gumley A McLeod H Lysaker PH. Metacognition and intersubjectivity: reconsidering their relationship following advances from the study of persons with psychosis. Front Psychol. (2020) 11:567. doi: 10.3389/fpsyg.2020.00567, PMID: 32269546 PMC7109331

[9] Lysaker PH Gagen E Moritz S Schweitzer RD. Metacognitive approaches to the treatment of psychosis: a comparison of four approaches. Psychol Res Behav Manag. (2018) 11:341–51. doi: 10.2147/PRBM.S146446, PMID: 30233262 PMC6130286

[10] Aydin O Balikci K Tas C Aydin PU Danaci AE Brüne M. The developmental origins of metacognitive deficits in schizophrenia. Psychiatry Res. (2016) 245:15–21. doi: 10.1016/j.psychres.2016.08.012, PMID: 27526312

[11] Frith CD. Schizophrenia and theory of mind. Psychol Med. (2004) 34:385–9. doi: 10.1017/s0033291703001326 15259823

[12] Lysaker PH Erickson M Ringer J Buck KD Semerari A Carcione A. Metacognition in schizophrenia: the relationship of mastery to coping, insight, self-esteem, social anxiety, and various facets of neurocognition. Br J Clin Psychol. (2011) 50:412–24. doi: 10.1111/j.2044-8260.2010.02003.x, PMID: 22003950

[13] Macbeth A Gumley A Schwannauer M Carcione A McLeod HJ Dimaggio G. Metacognition in first episode psychosis: item level analysis of associations with symptoms and engagement. Clin Psychol Psychother. (2016) 23:329–39. doi: 10.1002/cpp.1959, PMID: 25963712

[14] Arnon-Ribenfeld N Hasson-Ohayon I Lavidor M Atzil-Slonim D Lysaker PH. The association between metacognitive abilities and outcome measures among people with schizophrenia: a meta-analysis. Eur Psychiatry. (2017) 46:33–41. doi: 10.1016/j.eurpsy.2017.08.002, PMID: 28992534

[15] Salaminios G Debbané M. A mentalization-based treatment framework to support the recovery of the self in emerging psychosis during adolescence In: Hasson-Ohayon I Lysaker PH, editors. The recovery of the self in psychosis: contributions from metacognitive and mentalization based oriented psychotherapy. London: Routledge (2021). 12–35.

[16] Brent BK Holt DJ Keshavan MS Seidman LJ Fonagy P. Mentalization-based treatment for psychosis: linking an attachment-based model to the psychotherapy for impaired mental state understanding in people with psychotic disorders. Isr J Psychiatry Relat Sci. (2014) 51:17–24. PMID: 24858631

[17] Lempa G von Haebler D Montag C. Psychodynamische Psychotherapie der Schizophrenien: Ein Manual. Gießen: Psychosozial-Verlag (2016).

[18] Rosenbaum B Harder S Knudsen P Koster A Lindhardt A Lajer M. Supportive psychodynamic psychotherapy versus treatment as usual for first-episode psychosis: two-year outcome. Psychiatry. (2012) 75:331–41. doi: 10.1521/psyc.2012.75.4.331, PMID: 23244011

[19] Lysaker PH Buck KD Carcione A Procacci M Salvatore G Nicolò G. Addressing metacognitive capacity for self reflection in the psychotherapy for schizophrenia: a conceptual model of the key tasks and processes. Psychol Psychother. (2011) 84:58–69. doi: 10.1348/147608310X520436, PMID: 22903831

[20] Vohs JL Leonhardt BL James AV Francis MM Breier A Mehdiyoun N. Metacognitive reflection and insight therapy for early psychosis: a preliminary study of a novel integrative psychotherapy. Schizophr Res. (2018) 195:428–33. doi: 10.1016/j.schres.2017.10.041, PMID: 29108671

[21] Fuchs T. Selbst und schizophrenie. DZPhil. (2012) 60:887–901. doi: 10.1524/dzph.2012.0067

[22] Fuchs T De Haan S Ludwig M Martin L. Selbst- und Welterleben in der Schizophrenie. Stuttgart: Kohlhammer (2022).

[23] Henry M. L'essence de la manifestation. Paris: Presses Universitaires de France (1963).

[24] Gallagher S. Philosophical conceptions of the self: implications for cognitive science. Trends Cogn Sci. (2000) 4:14–21. doi: 10.1016/S1364-6613(99)01417-5, PMID: 10637618

[25] Heinz A Voss M Lawrie SM Mishara A Bauer M Gallinat J. Shall we really say goodbye to first rank symptoms? Eur Psychiatry. (2016) 37:8–13. doi: 10.1016/j.eurpsy.2016.04.010, PMID: 27429167

[26] Sass LA Parnas J. Schizophrenia, consciousness, and the self. Schizophr Bull. (2003) 29:427–44. doi: 10.1093/oxfordjournals.schbul.a007017, PMID: 14609238

[27] Merleau-Ponty M. The philosopher and his shadow. (original work published 1960) In: McLeary RCT, editor. Signs. Evanston, IL: Northwestern University Press (1964). 159–81.

[28] Stanghellini G. Embodiment and schizophrenia. World Psychiatry. (2009) 8:56–9. doi: 10.1002/j.2051-5545.2009.tb00212.x, PMID: 19293962 PMC2652898

[29] Stanghellini G Ballerini M Mancini M. Other persons: on the phenomenology of interpersonal experience in schizophrenia (ancillary article to EAWE domain 3). Psychopathology. (2017) 50:75–82. doi: 10.1159/000456037, PMID: 28273660

[30] Lacan J. Die Psychosen. Das Seminar Buch III (1955–1956). Quadriga: Weinheim und Berlin (1997).

[31] Federn P. Ichpsychologie und die Psychosen. Frankfurt: Suhrkamp (1956).

[32] Searles HF. Collected papers on schizophrenia and related subjects (1965). London: Karnac (1986).

[33] Mahler M Pine F Bergman A. The psychological birth of the human infant. New York: Basic Books (1975).

[34] Peciccia M Benedetti G. The splitting between separate and symbiotic stätes of the self in the psychodynamic of schizophrenia. Int Forum Psychoanal. (1996) 5:23–38. doi: 10.1080/08037069608412719

[35] Green MF Jimenez AM. Clinical observations and neuroscientific evidence tell a similar story: schizophrenia is a disorder of the self-other boundary. Schizophr Res. (2022) 242:45–8. doi: 10.1016/j.schres.2021.12.032 35027299

[36] Mentzos S. Psychodynamische Modelle in der Psychiatrie. Göttingen: Vandenhoeck & Ruprecht (1991).

[37] Shai D Laor Black A Spencer R Sleed M Baradon T Nolte T. Trust me! Parental embodied mentalizing predicts infant cognitive and language development in longitudinal follow-up. Front Psychol. (2022) 13:867134. doi: 10.3389/fpsyg.2022.867134 35992465 PMC9386006

[38] Mentzos S. Dilemmatische Gegensätze im Zentrum der Psychodynamik der Psychosen. Forum Psychoanal. (2015) 31:341–52. doi: 10.1007/s00451-015-0216-5

[39] Burnham DL Gladstone AI Gibson RW. Schizophrenia and the need-fear dilemma. New York: International Universities Press (1969).

[40] Mentzos S. Begründung des Bipolaritätsmodells der Psychosen und einige Erläuterungen zu deren neurobiologischer Dimension In: Mentzos S Münch A, editors. Reflexionen zu Aspekten einer Theorie der Psychosen. Göttingen: Vandenhoeck & Ruprecht (2012). 10–23.

[41] Lempa G. Eine psychoanalytische Theorie des schizophrenen Wahns. Forum Psychoanal. (2015) 31:353–74. doi: 10.1007/s00451-015-0215-6

[42] Buber M. Ich und Du. Leipzig: Insel-Verlag (1923).

[43] Stern DN Sander LW Nahum JP Harrison AM Lyons-Ruth K Morgan AC. Non-interpretive mechanisms in psychoanalytic therapy. The 'something more' than interpretation. The process of change study group. Int J Psychoanal. (1998) 79:903–21. 9871830

[44] Stern DN. Der Gegenwartsmoment. Veränderungsprozesse in Psychoanalyse, Psychotherapie und Alltag. Frankfurt am Main: Brandes & Apsel (2005).

[45] Morgan AC. Process of change study group BM. Moving along to things left undone. Infant Ment Health J. (1998) 19:324–32. doi: 10.1002/(SICI)1097-0355(199823)19:3<324::AID-IMHJ9>3.0.CO;2-L

[46] Stern DN Process of Change Study Group BM. The process of therapeutic change involving implicit knowledge: some implications of developmental observations for adult psychotherapy. Infant Ment Health J. (1998) 19:300–8. doi: 10.1002/(SICI)1097-0355(199823)19:3<300::AID-IMHJ5>3.0.CO;2-P

[47] Debbané M Nolte T. Mentalization-based therapy in the light of contemporary neuroscientific research In: Bateman A Fonagy P, editors. Handbook of mentalizing in mental health practice. 2nd ed. Washington, D.C.: American Psychiatric Association Publishing (2019). 21–35.

[48] Gallagher S Varga S. Social cognition and psychopathology: a critical overview. World Psychiatry. (2015) 14:5–14. doi: 10.1002/wps.20173, PMID: 25655144 PMC4329883

[49] Gallese V Ferri F. Psychopathology of the bodily self and the brain: the case of schizophrenia. Psychopathology. (2014) 47:357–64. doi: 10.1159/000365638 25359279

[50] Tanaka S. Intercorporeality as a theory of social cognition. Theory Psychol. (2015) 25:455–72. doi: 10.1177/0959354315583035, PMID: 28458467 PMC5390942

[51] Bion WR. Attention and interpretation. London: Tavistock Publications (1970).

[52] Der Wollenweber H. Handlungsdialog als Herausforderung und Chance in der Psychosentherapie In: Lempa G Troje E, editors. Vom monolog zum dialog: Forum der psychoanalytischen Psychosentherapie. Göttingen: Vandenhock and Ruprecht (2012)

[53] Heimann P. On counter-transference. Int J Psychoanal. (1950) 31:81–4.

[54] Orange D. Emotional understanding: Studies in psychoanalytic epistemology. New York: Guilford Press (1995).

[55] Stuke F Bröcker AL Bayer S Heinz A Bermpohl F Lempa G. Between a rock and a hard place: associations between Mentzos' "dilemma", self-reported interpersonal problems, and psychosocial functioning in individuals with non-affective psychoses. Clin Psychol Psychother. (2020) 27:528–41. doi: 10.1002/cpp.2437, PMID: 32100357

[56] Lombardi R. Mental models and language registers in the psychoanalysis of psychosis: an overview of a thirteen-year analysis. Int J Psychoanal. (2003) 84:843–63. doi: 10.1516/002075703768284614 13678492

[57] Lombardi R Pola M. The body, adolescence, and psychosis. Int J Psychoanal. (2010) 91:1419–44. doi: 10.1111/j.1745-8315.2010.00356.x 21133906

[58] Ferrari AB. La rete di contatto In: Ferrari AB Stella A, editors. L'alba del pensiero. [the dawn of thought]. Rom: Borla (1998). 139–51.

[59] Winnicott DW. Reifungsprozesse und fördernde Umwelt. München: Kindler (1965).

[60] Frith CD. The cognitive neuropsychology of schizophrenia. Hove: Lawrence Erlbaum Associates (1992).

[61] Fonagy P Gergely G Jurist EL Target M. Affect regulation, mentalization and the development of the self. New York: Other Press (2002).

[62] Debbané M Salaminios G Luyten P Badoud D Armando M Solida TA. Attachment, neurobiology, and mentalizing along the psychosis continuum. Front Hum Neurosci. (2016) 10:406. doi: 10.3389/fnhum.2016.00406, PMID: 27597820 PMC4992687

[63] Fonagy P Allison E. The role of mentalizing and epistemic trust in the therapeutic relationship. Psychotherapy. (2014) 51:372–80. doi: 10.1037/a0036505 24773092

[64] Shai D Belsky J. Parental embodied mentalizing: how the nonverbal dance between parents and infants predicts children's socio-emotional functioning. Attach Hum Dev. (2017) 19:191–219. doi: 10.1080/14616734.2016.1255653, PMID: 27852170

[65] Brent BK Fonagy P. A mentalization-based treatment approach to disturbances of social understanding in schizophrenia In: Lysaker PH Dimaggio G Brüne M, editors. Social cognition and metacognition in schizophrenia: Psychopathology and treatment approaches. London, Waltham, San Diego: Academic Press, Elsevier (2014). 245–59.

